# Immunohistochemical Detection of Microglia Using Iba-1 as a Marker in Health and Disease

**DOI:** 10.17691/stm2026.18.1.03

**Published:** 2026-02-27

**Authors:** V.V. Guselnikova, O.V. Kirik, D.A. Sufieva, V.A. Razenkova, A.A. Beketova, D.E. Korzhevskii

**Affiliations:** PhD, Head of Laboratory of Experimental Histology and Confocal Microscopy, I.P. Pavlov Department of Physiology; Institute of Experimental Medicine, 12 Academician Pavlov St., Saint Petersburg, 197022, Russia; Associate Professor, Department of Fundamental Problems of Medicine and Medical Technologies, Medical Institute; Saint Petersburg State University, 7–9 Universitetskaya Embankment, Saint Petersburg, 199034, Russia; PhD, Senior Researcher, Laboratory of Functional Morphology of the Central and Peripheral Nervous System, I.P. Pavlov Department of Physiology; Institute of Experimental Medicine, 12 Academician Pavlov St., Saint Petersburg, 197022, Russia; PhD, Senior Researcher, Laboratory of Experimental Histology and Confocal Microscopy, I.P. Pavlov Department of Physiology; Institute of Experimental Medicine, 12 Academician Pavlov St., Saint Petersburg, 197022, Russia; PhD, Researcher, Laboratory of Functional Morphology of the Central and Peripheral Nervous System, I.P. Pavlov Department of Physiology; Institute of Experimental Medicine, 12 Academician Pavlov St., Saint Petersburg, 197022, Russia; Laboratory Assistant Researcher, Laboratory of Functional Morphology of the Central and Peripheral Nervous System, I.P. Pavlov Department of Physiology; Institute of Experimental Medicine, 12 Academician Pavlov St., Saint Petersburg, 197022, Russia; MD, DSc, Professor of the Russian Academy of Sciences, Head of the Laboratory of Functional Morphology of the Central and Peripheral Nervous System, I.P. Pavlov Department of Physiology; Institute of Experimental Medicine, 12 Academician Pavlov St., Saint Petersburg, 197022, Russia

**Keywords:** microglia, Iba-1, ischemia, SHR, amyloid plaques, ontogenesis, immunohistochemistry

## Abstract

**The aim of the study** was to evaluate the possibilities and limitations of using the immunohistochemical detection of Iba-1 protein for morphofunctional analysis of microglia under different conditions.

**Materials and Methods:**

In the study we used brain samples from Wistar rats at different ages: 7 days of postnatal development (n=18), 14 days of postnatal development (n=18), 4–6 months (n=22); the brain samples from 3–6-month-old SHR (spontaneously hypertensive rats) male rats (n=4); human cerebral cortex samples (n=10). Rabbit polyclonal antibodies against calcium-binding protein Iba-1 (Biocare Medical, USA) were used to detect microglia immunohistochemically followed by light and confocal laser microscopy. A previously developed original technique was applied to simultaneously detect microglial cells and amyloid plaques.

**Results:**

Iba-1 protein in microglial cells was shown to be present in grey and white matter in all cerebral regions under study. Microglial cells in different regions were noted to be characterized by pronounced structural and functional features when stained for Iba-1. In addition to microglial cells, in the rat brain there were found other Iba-1-immunopositive macrophages, which were tissue cerebral macrophages differing from microgliocytes by specific morphology and localization. Iba-1 protein was demonstrated to be present in microglial cells in all investigated stages of postnatal development that makes Iba-1 an appropriate marker for comparative ontogenetical studies of microglial cells. The localization of Iba-1 in the bodies and processes of microgliocytes enables to more clearly identify the complex ramified cell morphology and make three-dimensional reconstructions. In the brain of spontaneously hypertensive rats (SHR line rats) the Iba-1-immunopositive microglia were shown to have a number of structural and functional features indicating the moderate activation of microglia. 48 h after ischemic injury, an anomalously great number of large Iba-1-immunopositive cells with amoeboid morphology were detected in the gray matter of the striatum in an ipsilateral hemisphere near the injured area in the rat brain. Microglial cells in human cerebral cortex were noted to be localized in the majority of the detected amyloid plaques and characterized by amoeboid morphology indicating their severe activation.

**Conclusion:**

The presented findings suggest Iba-1 protein to be a reliable and universal microglial marker. The immunohistochemical detection of the protein enables to identify and analyze qualitatively and quantitatively microglial cells in different brain regions in human and laboratory animals under normal and pathological conditions. The limitations of using Iba-1 as a marker of microglia include the inability to determine the microglial activation vector and the difficulty in distinguishing between microglia and infiltrating macrophages of the brain in pathology. In such cases, it is necessary to improve the technology for detecting microglia that can be based on using a multi-marker analysis.

## Introduction

Currently, special attention of researchers in neurobiology is paid to the study of microglia, it is related to their multifunctionality and considering their leading role in developing neuroinflammation — a key factor of neurodegeneration [1, 2]. Intensive study of microglia makes it particularly topical to have in researchers’ arsenal a reliable technology to identify these cells in physiological and pathological conditions. The difficulty is that microglia are a highly heterogeneous population [3–6]. There have been shown the interspecies heterogeneity of microglia [7, 8], as well as sexual and regional heterogeneity of this cell population [5, 8, 9]. Moreover, microglia are highly dynamic and can easily change a gene expression pattern during ontogenesis and under pathological conditions [7, 8, 10, 11]. Microglial cells were demonstrated to express over 1000 receptor systems that makes them most sensitive to the factors of local microenvironment [3, 7, 12]. Due to such high sensitivity, microglia cells are able to quickly change their structural functional condition [13].

Among the modern technologies used to detect microglia, the most widely popular one to identify these cells is immunohistochemistry using ionized calcium-binding adapter molecule 1 (Iba-1) [5, 14, 15]. However, considering the marked heterogeneity and the dynamic quality of microglia, the question on using Iba-1 as a marker is urgent.

**The aim of the study** was to evaluate the applicability and limitations of using the immunohistochemical detection of Iba-1 protein for morphofunctional analysis of microglia under different conditions.

## Materials and Methods

The study was carried out in accordance with the Helsinki Declaration (2024), and approved by the local ethics committee of the Institute of Experimental Medicine (Russia) (protocol No.3/17 dated November 30, 2017, protocol No.3/18 dated November 22, 2018, protocol No.1/22 February 18, 2022).

As the study, material we used Wistar rat cerebral samples of various age groups: day 7 of postnatal ontogenesis (n=18); day 14 of postnatal ontogenesis (n=18); rat adults aged 4–6 months (n=22); the cerebral samples from male SHR (spontaneously hypertensive rats) aged 3–6 months (n=4); and human cerebral cortex samples (n=10).

The young rats with the dated date of birth were obtained according to the recommendations by Dyban et al. [16]. Transient focal ischemia was modeled according to the previously described technique [17]. To study human cerebral samples, we used the archive autopsy material previously obtained [18] — 10 human cerebral cortical samples (males, n=2 and females, n=8; aged from 65 to 94) with amyloid plaques. The preservation of the material and its applicability for immunohistochemistry were determined using a standard test immunohistochemical reaction to glial fibrillar acid protein.

Human brain samples were fixed in 10% buffered formalin, and rat brain samples — in zinc-ethanol-formaldehyde [19]. After fixation, the material was dehydrated and embedded in paraffin followed by making the sections, 5 μm thick, and arranging on adhesive slides (Menzel, Germany).

For immunohistochemical identification of microglia, we used rabbit polyclonal antibodies against Iba-1 at a dilution of 1:1000 (Biocare Medical, USA). The components from the kit — Reveal Rabbit Specific HRP-DAB Detection System (Spring Bioscience, USA) were applied as the secondary reagents. For the reaction product imaging, we used chromogen 3,3’-diaminobensidine from the kit: DAB+ (Agilent, USA). After the reaction, some sections were counterstained with alum hematoxylin.

For simultaneous detection of amyloid plaques and microglia cells, the human cerebral cortical sections were treated applying an original technique based on immunohistochemical detection of microglia using the antibodies against Iba-1, and histochemical alcian blue staining of amyloid plaques [20].

The sections were analyzed on the light microscope Leica DM750 (Leica Microsystems, Germany), and the photos were taken using the camera ICC50 (Leica Microsystems, Germany) and the software program LAS EZ (Leica Microsystems, Germany).

For immunofluorescent analysis of microglia, we used rabbit polyclonal antibodies against Iba-1 at a dilution of 1:500 (Biocare Medical, USA). The primary antibodies were revealed using the monovalent Fab-fragment of antirabbit donkey immunoglobulin conjugated with fluorochrome Rhodamine Red™-X (RRX) (Jackson ImmunoResearch, USA). The nuclei were stained with fluorescent nuclear stain Sytox Green (Invitrogen, USA). The obtained sections were analyzed on a confocal laser microscope Zeiss LSM 800 (Zeiss, Germany). To excite RRX fluorescence we used a 561-nm-wavelength laser, for Sytox Green — 488 nm; and the lens: Plan-Apochromat 20×/0.8 M27 and Plan-Apochromat 63×/1.40 Oil DICM27 (oil immersion). The findings were studied using the software ZEN 2.6 blue edition (Zeiss, Germany).

## Results

### Using the Iba-1 marker to study the structural and functional state of microglia and its regional features

**Figure 1.**
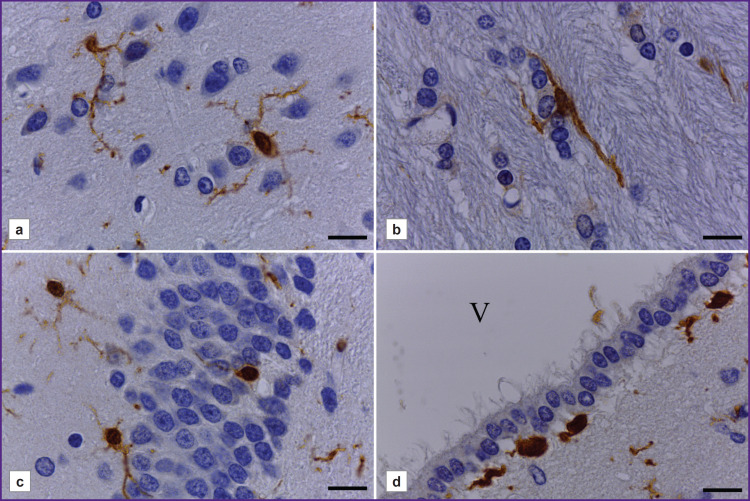
Morphological characteristics of microglia in different regions of the rat brain: (a) cerebral cortex; (b) subcortical white matter; (c) hippocampal dentate gyrus; (d) subependymal zone of III ventricle. Immunohistochemical reaction to Iba-1 with alum hematoxylin counterstaining. V — III ventricular cavity. Bar — 20 μm

The analysis of brain sections from adult rats of all studied samples showed the presence of Iba-1-immunopositive cells in all areas of the brain included in the section plane. An immunohistochemical reaction was characterized by high intensity and the absence of background staining. Iba-1-immunopositive cells in the cerebral cortex, subcortical white matter, striatum, hippocampus, and other subcortical structures of the endbrain had a typical ramified microglia morphology (Figure 1). Iba-1 was present in the body and the processes of microgliocytes.

There was found the morphological diversity of Iba-1-immunopositive cells depending on their localization in different cerebral regions. In the cerebral cortex there were microgliocytes of small soma (round or oval) with long thin processes, highly branching into different directions (Figure 1 (a)).

Microglial cells in the subcortical white matter were fusiform (Figure 1 (b)). They were characterized by the presence of one or two (rarely — three) long, non-branching or weakly branching processes extending from different cell poles in opposite directions. The bodies and the processes of microglial cells were orienting along the nerve fibers (see Figure 1 (b)).

Microglia in the hippocampus had the morphology similar to that of microglia in the cerebral cortex, and was characterized by the presence of a small soma with small-volume perinuclear cytoplasm and numerous thin branching processes (Figure 1 (c)). Microglial processes were running through all hippocampal neuropil and penetrating the neuronal layers of the hippocampus. The bodies of microgliocytes, as a rule, were revealed beyond the neuronal layers, and frequently — they bordered closely on them. Occasionally, the bodies of microgliocytes were found directly as part of a pyramid layer or a granular layer of the dentate gyrus, they were being located between the neurosomes (see Figure 1 (c)). In the subependymal zone of the brain ventricles (lateral, III), the microgliocytes had ameboid morphology — they are the cells with a rounded/ elongated soma and short non-branching processes, or without processes (Figure 1 (d)). Some fusiform cells formed some processes, which were directed to the underlying nerve tissue or a layer of ependymal cells.

**Figure 2.**
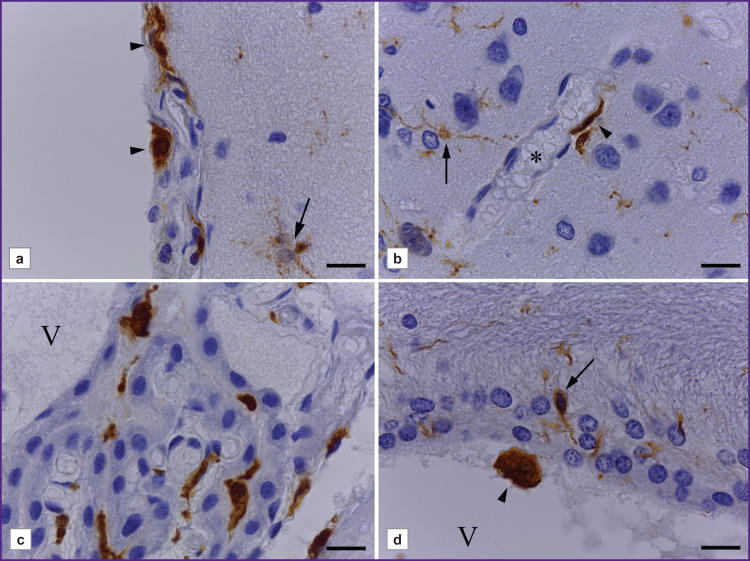
Resident macrophages in the rat brain: (a) meningeal macrophages; (b) perivascular macrophages; (c) Kolmer cells and stromal cells of choroid plexus; (d) supraependymal macrophage in the lateral ventricle. Immunohistochemical reaction to Iba-1 with alum hematoxylin counterstaining. V — ventricular cavity. The vascular lumen is indicated by an asterisk, microgliocytes — by arrows, macrophages — by arrow heads. Bar — 20 μm

Alongside microglial cells, the rat brain was observed to have other Iba-1-immunopositive cells, which were cerebral tissue macrophages (Figure 2). In the cerebral meninges we revealed meningeal macrophages — branchless round or oval cells, rarer — fusiform cells with an intensively stained cytoplasm (Figure 2 (a)). In different cerebral regions near blood vessels there were revealed perivascular Iba-1-immunopositive cells with few processes, the cells being spread along the capillary axis, they were perivascular macrophages (Figure 2 (b)). In the choroid plexus there were Kolmer cells, which had an oval-shaped soma, and formed no processes or had short non-branched processes. In the connective tissue of choroid plexus stroma, around the vessels, there were elongated Iba-1-immunopositive cells (Figure 2 (c)). On the ependymal surface there were single supra-ependymal macrophages — oval, without processes, with an intensive reaction of the cytoplasm to Iba-1 antigen (Figure 2 (d)).

### Using the Iba-1 marker for the comparative ontogenetic study of microglia

Using confocal laser microscopy, we studied rat striatal microgliocytes at different stages of postnatal ontogenesis (P7, P14, adult animals). Iba-1 protein was present in the bodies and the processes of microgliocytes at all ontogenesis stages under study.

**Figure 3.**
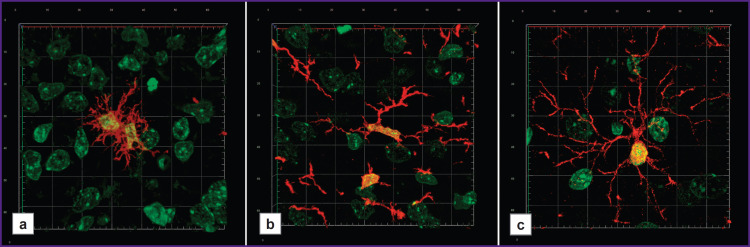
Morphological characteristics of microglia in the rat striatum at different stages of postnatal ontogenesis: (a) day 7 of postnatal development; (b) day 14 of postnatal development; (c) adult animal. Immunofluorescent reaction to Iba-1 (*red color*), the nuclei of cells are stained with Sytox Green (*green color*). Confocal laser microscopy, three-dimensional reconstruction. Grid cell size — 10×10 μm

On day 7 of postnatal development, the microglia were characterized by a variety of morphotypes: some microgliocytes had wide blade-like cytoplasmic processes, others — 2–3 large non-branching processes; there were also the microgliocytes with a great number of short thin branching processes (Figure 3 (a)). All detected microgliocytes at this postnatal stage were small-sized.

On day 14 of postnatal development, the majority of detected Iba-1-immunopositive cells had small, frequently — elongated body with several long, relatively thin, moderately branching processes (Figure 3 (b)).

Striatal microglia in adults had morphological uniformity: all detected microgliocytes had a small body and numerous thin, multibranch processes extending away from the cell body to different directions (Figure 3 (c)).

### Using the Iba-1 marker to study microglia in the nervous system pathology

**Figure 4.**
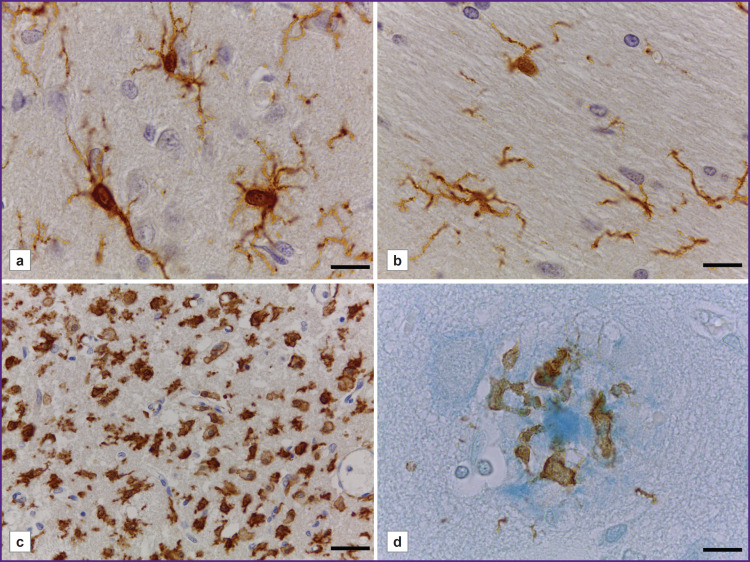
Activated microglial types in pathology: (a, b) moderate activation of microglia in the cerebral cortex (a) and the white matter (b) of the brain in SHR rats; (c) strong microglial activation in the ischemic injury area in the rat brain; (d) ameboid microglia as part of an amyloid plaque in human cerebral cortex. Immunohistochemical reaction to Iba-1; (a–c) counterstaining with alum hematoxylin; (d) counterstaining with alcian blue. Bar — 20 μm (a, b, d) and 50 μm (c)

The microglia in the brain of spontaneously hypertensive rats (SHR) (Figure 4 (a, b)) had a number of structural and functional features when immunostained with anti-Iba-1 antibody: there was an increase in the size of microglial cell bodies, as well as the thickening of their processes, which had intensive branching in both: grey (see Figure 4 (a)) and white matter (Figure 4 (b)).

48 h after an ischemic injury, in the rat brain there was revealed an anomalously great number of large Iba-1-immunopositive cells with ameboid morphology in the grey matter of striatum in an ipsilateral hemisphere near the injured area (Figure 4 (c)). They were characterized by rounded or ameboid shape, no processes. Some cells had only small cytoplasmic processes (see Figure 4 (c)). It seems to be impossible to determine the belonging of the cells to microglia or macrophages. There were no microglia with normally branched morphology in the ischemic injury area.

In human cerebral cortex, there were microglial cells as part of most amyloid plaques revealed when using alcian blue (Figure 4 (d)). Microglial cells had ameboid morphology: their bodies were large, irregularly-shaped; occasionally there were short cytoplasmic processes with bulbous thickenings. Microglial cells were located within a fibrous halo surrounding the dense core of the plaque. Microglial processes could extend and closely adjoin the central dense core, and there were no Iba-1-immunopositive structures inside the central part (see Figure 4 (d)). Microglial immunostaining intensity in this case was significantly lower compared with the intensity of the immunohistochemical reaction to Iba-1 in rats.

## Discussion

Within the framework of the present study, we evaluated the possibilities and limitations of using the immunohistochemical detection of Iba-1 protein for a morphofunctional analysis of microglia under different conditions. Iba-1 protein was demonstrated to be present in the microglial cells in all cerebral regions under study, in grey and white matter, in adult Wistar rats. In addition, in different regions, microglial cells were characterized by morphological features, and that was in good agreement with the current concept of high regional heterogeneity of this cell population [9]. We found no differences in the immunohistochemical reaction intensity to Iba-1 in different cerebral regions. From this point of view, Iba-1 can be considered as a universal marker to study microglia in different cerebral regions, regardless high regional specificity typical for these cells. The advantage of Iba-1 as a microglial marker is also the high conservation of the protein that enables to use the antibodies against Iba-1 to detect microglia in different laboratory animals and human [21]. Iba-1 is a cytoplasmic protein present both in the body and the process of microgliocytes [7, 15]. Due to the cytoplasmic localization and uniform distribution of Iba-1 inside a cell, immunohistochemical detection of the protein enabled to reveal most completely the complex structure of microgliocyte processes, as well make 3D-reconstructions of the cells; it enables to apply immunohistochemical reaction to Iba-1 as a tool for the most thorough study of microglial morphology. Other widely used proteins — the microglial markers (CD68, P2Y12R, TMEM119) [5, 7, 22, 23] are less suitable for the purpose, since they are transmembrane molecules, and frequently have “dashed” distribution. Their application as immunohistochemical markers fails to assess to the full extent the peculiarities of the complex morphology of microglial cells.

When studying the microglia in the rat striatum at different postnatal ontogenesis stages, we succeeded in demonstrating Iba-1 to be a suitable marker to study the dynamic pattern of microglial cell morphology. Based on literature data, Iba-1 starts expressing in progenitor microglial cells at early embryogenesis [24], and is preserved within the entire following ontogenesis; it makes it possible to carry out microglial comparative ontogenetic studies using Iba-1 as a marker. It enables to distinguish Iba-1 from many other marker proteins of microglia. For instance, there is no TMEM119 in immature microglia in a prenatal and an early (up to P14) postnatal period [15].

The present study findings along with numerous literature reports [23] indicate that Iba-1 protein is present in microglial cells of a various activation degree. Microglia are usually subdivided into two morphofunctional types: branched (with processes) and ameboid. Microglia with processes is a “resting” microglial form (according to an old terminology), which has lately been called “surveillance”. These cells are characterized by a small body, and the presence of long thin branching processes, which are in constant motion: they are able to quickly get longer or shorter, form small temporary filopodia. Ramified microglia are considered to be incapable to phagocytosis, and the main function of these cells is continuous scanning of the local microenvironment for pathological stimuli, and the interaction with other cells and extracellular components of the nervous tissue [5]. In response to damage, microglia quickly change their molecular and structural properties, which affects their morphology — the cell body increases in size, the processes shorten and thicken [5, 23]. A terminal stage of microglial activation is its ameboid form — high-mobility phagocytic cells participating in the antigen presentation [5, 15]. The data we obtained by studying different pathological types suggest Iba-1 to be present in both: branched and ameboid microglia, as well as in all intermediate forms. It makes the Iba-1 protein a convenient tool to study microglia in health and disease, and enables to evaluate and differentiate different pathological conditions. So, in Iba-1-immunostaining on the rat brain sections after transient middle cerebral artery occlusion we had the representative changes of microglia in an ischemic injury previously described [25–27]: the staining intensity and the density of Iba-1-immunopositve elements significantly increased in the ischemic core and in the “ischemic penumbra” area compared to the intact hemisphere, and the Iba-1-positive cells had a specific ameboid or rounded shape. The literature data have also indicated an increased Iba-1 protein expression in the ischemic core and the adjoining area [28].

When studying human cerebral cortex samples with amyloid plaques, we observed a typical morphology. In this case, the activated microglial cells were revealed to closely surround amyloid plaques. Microglial activation and its clustering around amyloid plaques is a well-known phenomenon, however, the true role of microglia in this case remains unclear. On the one hand, microglia were shown to block the toxic effect of beta-amyloid by forming a separating barrier around amyloid plagues [29]. Moreover, microglia are able to phagocyte amyloid fibrils [30]. On the other hand, continuous microglial activation causes chronic neuroinflammation resulting in neuronal damage [31]. The study by Bubnova et al. [32] carried out on postmortem cerebral samples of patients with Alzheimer’s disease demonstrated an increase in Iba-1 protein expression manifested in different extents depending on the disease stage. It is interesting to note that the intensity of Iba-1-immunostaining of microglia was decreased associated with amyloid plaques in human cerebral cortex. It could be resulted from the microglial phenotype changing into ameboid. Although it is not inconceivable that the Iba-1 expression level in microglia associated with amyloid accumulations decreases at certain disease stages leading to Iba-1 protein content decrease in these cells.

In addition to numerous advantages of immunohistochemical detection of Iba-1 to study microglia, the method has a number of limitations, which should be taken into consideration when planning research. So, in world scientific literature there are the reports on microglial cells losing Iba-1 expression in some cerebral regions when developing such conditions as schizophrenia, Huntington’s disease, obesity [9, 23, 33]. In the mentioned cases, the use of Iba-1 as a single marker fails to completely detect the microglial population.

Additionally, the use of Iba-1 fails to determine a microglial activation vector (M1/anti-inflammatory or M2/ anti-inflammatory) that makes it impossible to reveal the exact functional contribution of microglia into certain pathology.

One more significant restriction of using Iba-1 is the presence of this protein in microglial cells, as well as in cerebral tissue macrophages and in peripheral macrophages [5, 23, 34]. The studies we carried out demonstrated that in health microglia can be distinguished from tissue macrophages in response to Iba-1 by the localization and morphology features. However, in pathology, it can be challenging to distinguish the activated ameboid microglia from recruited macrophages (blood monocyte progenitors). So, we found that following temporary middle cerebral artery occlusion in rats, the density and intensity of Iba-1 staining were significantly higher in the ischemic core and the ischemic watershed compared with the intact hemisphere. The ischemic injury zone had a great number of Iba-1-immunopositive cells of ameboid shape. In this case, it seems to be impossible to determine if the cells are activated microglia or recruited macrophages. In such cases, the use of Iba-1 as a single marker to study microglia is likely to be insufficient. The technology improvement feasibility is the simultaneous usage of several marker proteins. Currently, the most promising for these purposes is the use of relatively recently described proteins TMEM119 and P2RY12. TMEM119 is a transmembrane protein consisting of 283 amino acids; it is present only on the surface of microglial cells and not expressed in other monocyte-macrophage cells [22]. Purinergic receptor P2RY12 conjugated with G-protein is also highly specific for microglia [6, 11, 35, 36]. Both proteins were characterized as homeostatic microglial markers demonstrating decreased expression in the activation of microglial cells [11, 22, 37, 38]. The application of the combination of these markers with Iba-1, the expression of which, on the contrary, increases in activation [5, 23], is sure to enable to thoroughly and highly specifically detect microglial population in health and disease. The development of proper protocols of double and/or triple marking is considered an important methodological challenge of modern neurobiology.

## Conclusion

Iba-1 can be considered a reliable and universal microglial marker. Iba-1-immunostaining enables to identify and analyze (qualitatively and quantitatively) microglia in different cerebral regions in human and laboratory animals in health and disease. The limitations of using Iba-1 as a marker of microglia are related primarily to the inability to determine a microglial activation vector, as well as to the difficulty in distinguishing microglia from infiltrating cerebral macrophages in pathology. In such cases, it is necessary to improve the technology of microglia detection, it can be based on applying a multi-marker analysis.
